# Deletion of the V2 vasopressin receptor gene in two Chinese patients with nephrogenic diabetes insipidus

**DOI:** 10.1186/1471-2156-7-53

**Published:** 2006-11-14

**Authors:** Yan Dong, Haihui Sheng, Xueru Chen, Jun Yin, Qing Su

**Affiliations:** 1 Department of Endocrinology, Xinhua Hospital, Shanghai Jiaotong University School of Medicine, Shanghai 200092, China; 2 National Engineering Center for Biochip at Shanghai, Shanghai 201203, China

## Abstract

**Background:**

Congenital nephrogenic diabetes insipidus (NDI) is a rare X-linked inherited disorder characterized by the excretion of large volumes of diluted urine and caused by mutations in arginine vasopressin receptor 2 (AVPR2) gene. To investigate the mutation of AVPR2 gene in a Chinese family with congenital NDI, we screened AVPR2 gene in two NDI patients and eight family members by PCR amplification and direct sequencing.

**Results:**

Five specific fragments, covering entire coding sequence and their flanking intronic sequences of AVPR2 gene, were not observed in both patients, while those fragments were all detected in the control subjects. Several different fragments around the AVPR2 locus were amplified step by step. It was revealed that a genomic fragment of 5,995-bp, which contained the entire AVPR2 gene and the last exon (exon 22) of the C1 gene, was deleted and a 3-bp (GAG) was inserted. Examination of the other family members showed that the mothers and the grandmother were carriers for this deletion.

**Conclusion:**

Our findings suggest that the two patients in a Chinese family suffering from congenital NDI had a 5,995-bp deletion and 3-bp (GAG) insertion at Xq28. The deletion contained the entire AVPR2 gene and exon 22 of the C1 gene.

## Background

Congenital nephrogenic diabetes insipidus (NDI) is a rare inherited disorder characterized by the excretion of large volumes of diluted urine resulting from resistance of the renal collecting duct to the antidiuretic hormone arginine vasopressin (AVP), and the clinical characteristics include polyuria, polydipsia, fever, hypernatremic dehydration, and constipation. Ninety percent of congenital NDI are linked to the X-chromosome and are caused by loss-of-function mutations in the arginine vasopressin type 2 receptor (AVPR2) gene. Since human AVPR2 gene was cloned successfully in 1992, more than 190 distinct disease-causing mutations in AVPR2 gene have been identified in X-linked congenital NDI families[1]. The mutations consist of missense, nonsense, frameshift, splice-site, insertion, and deletion mutations. These mutations are not concentrated on one domain of the receptor, but are scattered throughout the protein, except for the part coding of the N- and C-terminal tails of the receptor. The phenomenon of so many mutations in AVPR2 gene in NDI patients indicates that this disease is highly heterogeneous at the molecular level. Among these mutations, the vast majority result in single amino acid substitutions (missense mutations) or receptor truncations caused by nonsense or frameshift mutations[1]. In addition to these mutations, there were some reports describing the total deletion of the AVPR2 gene [2-7], however, the precise identification of the breakpoints of the fragment deletion was done in only three reports[4,6,7].

In this study, we ascertained two patients suffering from congenital NDI in a Chinese family caused by a 5,995-bp genomic fragment deletion and 3-bp insertion at Xq28, and this deletion encompassed a complete loss of the AVPR2 gene and the last exon (exon 22) of the C1 gene, which is adjacent closely to the AVPR2 gene locus.

## Results

The two patients in this Chinese NDI family showed normal statures, the proband (III-1) was 174 cm height and 80 kg weight, and his young cousin (III-2) was 170 cm height and 70 kg weight, respectively. Neither of them had any evidence of mental retardation or significant diseases other than NDI. All chemical laboratory tests were performed using standard clinical laboratory assays. The chemical laboratory tests including blood glucose, liver function, renal function, triglyceride, total cholesterol, blood electrolyte (potassium, sodium, calcium, phosphorus), coagulation parameters (prothrombin time, partial-throboplastin time) and hemotology (red blood cell count, hemoglobin, white blood cell count, and platelet count) were all within reference range (data not shown). There were no abnormality of electrocardiographic measurements and no abnormality of pituitary by Magnetic Resonance Imaging (MRI) examinations, and the ultrasonographic examinations of urinary system revealed that there were normalities in size and structure of kidney, ureter and urinary bladder.

**Figure 1:**
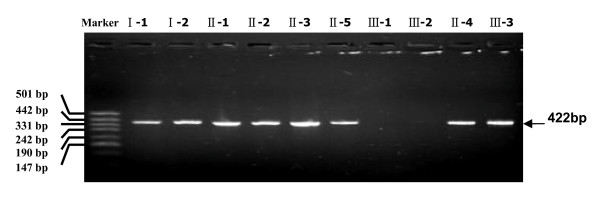
**PCR amplification of the coding region of the AVPR2 gene**. Five specific fragments covering the AVPR2 gene were amplified (see Methods). One example of the different PCR products, which was emplified by the primer pair 1F/1R, is shown exemplarily. Only in the control subjects (I-1, I-2, II-1, II-2, II-3, II-4, II-5 and III-3) the specific fragments were detected.

Genomic DNAs from the NDI patients and the related family members were prepared and subjected to PCR amplification (five overlapping DNA segments) of the entire coding regions of AVPR2 gene (GenBank accession number NM_000054). PCRs with all five primer pairs within the AVPR2 locus yielded five specific fragments of the expected sizes (422 bp, 480 bp, 450 bp, 476 bp, and 470 bp) for the control subjects, the sequences of these five fragments were the same as those from GenBank (Figure 1). However, no corresponding PCR product yielded with these five primer pairs was observed in the two patients. In addition, we performed PCR for the PCK1 gene and detected the expected product in all NDI patients, indicating that PCR amplification was effective and reliable in every sample (data not shown) and the entire AVPR2 gene may be deleted in these two patients.

**Figure 2:**
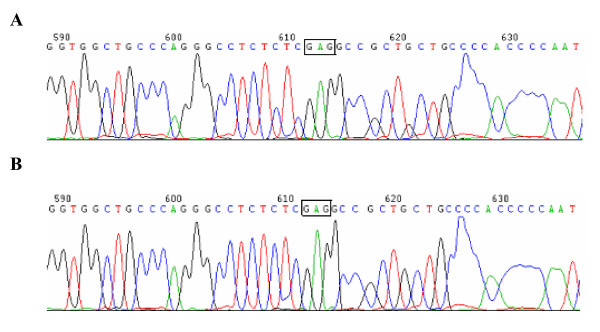
**The sequence analysis of the deletion breakpoints in two patients**. A and B represent the sequence of the proband (III-1) and the other patient (III-2), respectively. The 3-bp insertion (GAG) is boxed. The 5' breakpoint was upstream of the GAG-insertion, while the 3' breakpoint was downstream of this insertion. Sequence alignment with Genbank accession number U52112 and NM_001666 revealed a deletion of a 5,995-bp fragment.

**Figure 3:**
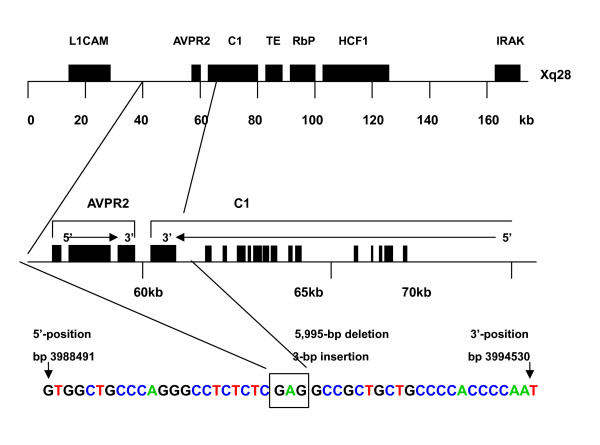
**Model of the AVPR2 gene with the chromosomal map of a submicroscopic deletion found in the patients described**. The location of the submicroscopic deletion within the q28 region of the chromosome X between the L1CAM and C1 genes loci is shown. The upstream breakpoint was identified within the intergenic region between L1CAM and AVPR2 genes, while the downstream breakpoint was identified within the intron 21 of the C1 gene by PCR screening, resulting in deletion of the whole AVPR2 gene and the last exon (exon22) of the C1 gene. To identify the exact positions of the deletion breakpoints, a primer pair 7F/9R was used to amplify interstitial genomic sequence. The 1160-bp fragment was obtained and sequenced.

In order to map the deletion breakpoint, we investigated the presence or absence of several different fragments around the AVPR2 locus step by step. In these two patients, with primer pair 7F/7R but not 6F/6R a PCR product was amplified, suggesting the 5' breakpoint in the region between primers 6R and 7R. Using the similar strategy, the 3' breakpoint was suspected to be located in region between sequences of primers 8F and 9F in the AVPR2 gene. The deletion junction was finally amplified with the primer pair 7F/9R, giving an approximately 1.1 kb PCR product. The 1.1 kb PCR product, which contained the deletion breakpoint, was then sequenced in both directions. The sequence alignment revealed that a genomic fragment of 5995-bp was deleted and three bases (GAG) were inserted (Figure 2-A and 2-B). Therefore, the 5' breakpoint was located within intergenic region between L1CAM gene (GenBank accession number U52112) and AVPR2 gene, while the 3' breakpoint was located within intron 21 of the C1 gene (GenBank accession number NM_001666). The deletion region contained the entire AVPR2 gene and the part of the C1 gene from intron 21 to exon 22 (Figure 3).

**Figure 4:**
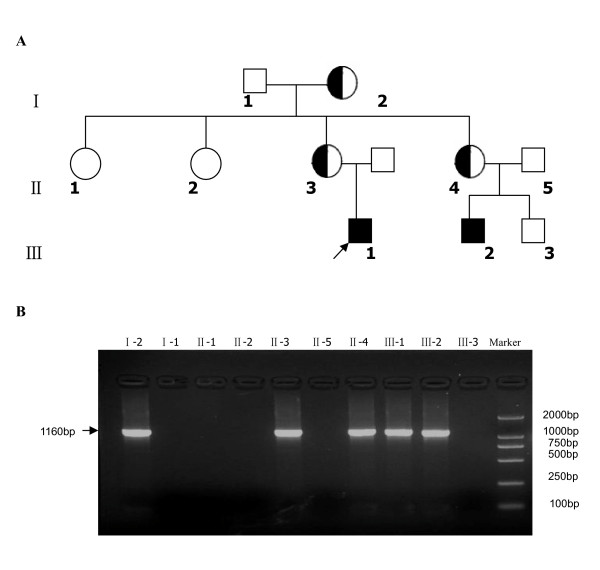
**(A) The pedigree of a Chinese family with congenital NDI**. (The proband is indicated by an arrow); **(B) Amplification of the mutated allele in family members**. The 1160 bp fragment for the presence of the deletion of the AVPR2 gene was amplified with the primer pair of 7F/9R. The fragments were observed in number I-2, II-3, II-4, III-1 and III-2, suggesting that number I-2, II-3 and II-4 were heterozygous for the same fragment deletion and GAG insertion mutation on X chromosome.

In order to investigate whether a family member had the same deletion, their genomic DNAs were used as amplification templates in a single-plex reaction using the primer pair 7F/9R. The specific fragments of expected length (1160 bp) were observed in the mothers of both patients (II-3 and II-4) and the grandmother (I-2), whereas the corresponding fragments were not yield in the other family members in this NDI pedigree(Figure 4B). These results showed that the mothers and the grandmother were heterozygous for the same fragment deletion and GAG insertion mutation on X chromosome.

## Discussion

With the development of microarray technology, numerous copy number variations are identified in the human genome[8]. Copy number variation is associated with many normal phenotypic variations and diseases[9]. Most copy number variations involved diseases are originally found in Mendelian diseases. In the present study, we have identified a novel type mutation of 5,995-bp deletion and 3-bp insertion, which leaded to a complete loss of the AVPR2 gene and the last exon (exon 22) of the C1 gene, in an X-linked congenital NDI family.

The AVPR2 receptor, which is composed of 371 amino acids, consists of seven transmembrane domains, four extracellular domains and four cytoplasmic domains, and belongs to the superfamily of G protein-coupled receptors (GPCRs) [10-12]. On binding to AVP, the receptor activates the Gs/adenylate cyclase, and this leads to increased level of the intracellular cyclic adenosine monophosphate (cAMP). The elevated cAMP activates protein kinase A and starts a phosphorylation cascade that promotes the translocation of the water channel, aquaporin 2, to the apical membrane of the renal tubules and collecting tubules. This results in increasing water resorption[13]. Therefore, the person will present polyuria if he has abnormal function or loss-of-function of AVPR2. In our study, the two patients suffered from NDI were caused by the complete deletion of AVPR2 gene. The cause of this genomic fragment deletion on chromosome may be the abundant repetitive DNA in the human genome, which is commonly suggested that microdeletions arise through mispairing of large duplicated sequences[14].

In our report, besides the entire loss of the AVPR2 gene, the last exon (exon 22) and part of intron 21 of the C1 gene were also deleted in these two patients. The C1 gene is in close proximity to the AVPR2 locus and is orientated in opposite direction to the reading frame of the AVPR2 gene. The C1 gene, being highly expressed in hematopoietic cells, encodes a protein that appears to represent a member of the increasingly large group of signaling proteins involved in regulation of small GTP binding proteins of ras superfamily[15]. Based on the domain and functional data, it is suggested that the protein encoded by C1 gene acts as a Rho-GTPase-activating protein (ARHGAP4)[13], belonging to the large superfamily of GTPase-activating proteins. The genomic defect in the C1 gene in our study locates the C-terminal portion of ARHGAP4, which includes consensus sites of transcription termination (polyadenylation signals) and consensus sites for mRNA stability. This, at least theoretically, in a new C terminus due to a frameshift[7]. Consequently, it was reasonable to suppose that ARHGAP4 expression and function might be altered in these two patients. However, the two patients in our study did not present any major abnormalities besides clearly defined NDI symptoms caused by deletion of the entire AVPR2 gene. Their electrocardiographic measurements, various laboratory tests in clinical chemistry and hematology were repeatedly normal. Coagulation parameters were also within the reference ranges. These clinical data were similar to the patients described previously[4,7]. The reason was postulated that the lack of ARHGAP4 function was most likely compensated by other members of the GAP family[4]. In addition to the mentioned above, the two patients in our study showed normal statures and intelligence and had normal size and structure in kidney, ureter and bladder, while one patient reported by Schoneberg T showed mental retardation, reducing reading and writing skills[4], and the other patient reported by Schulz et al. presented an enlarged bladder and ureter dilatation[7]. The difference between them might be associated with the degree of polyuria and hypernatremic dehydration.

As to the female carriers, they are usually phenotypically normal because of possessing two X chromosomes. However, approximately 1% of females heterozygous for an AVPR2 gene mutation may display variable degrees of polyuria and polydipsia, which has been explained by a preferential inactivation (skewing) of the X chromosome bearing the normal AVPR2 allele. Three heterozygote females in this kindred of NDI have no symptom of polyuria and polydipsia.

## Conclusion

We have identified a novel type of 5,995-bp deletion and 3-bp (GAG) insertion at Xq28 in two Chinese patients with NDI, which including the complete loss of the AVPR2 gene and the last exon (exon 22) of the C1 gene, leading to NDI without other symptoms.

## Methods

### Patients Profile

The pedigree of this Chinese family with congenital NDI is shown in Figure 4A. Two patients with congenital NDI and eight normal persons belonging to this family were investigated.

The proband (III-1), who was 20 years old, was admitted to our department for investigation of polydipsia and polyuria. He was a full-term infant and was delivered uneventfully. His parents were unrelated and healthy and had no history of inheritable diseases. The proband had presented with severe polydipsia and polyuria since 7 months of age, and he had not been diagnosed and treated until the age of 20 years old. When he was admitted to our department, he passed 7000~10900 ml urine per day and the specific gravity of urine was between 1.000 and 1.005. NDI was diagnosed basing on clinical symptoms, laboratory findings, and failure to increase urine volume and urinary osmolality after administration of 5 u vasopressin (Table 1). The urine volume was reduced about 50% by hydrochlorothiazide treatment.

**Table 1:** The results of combined water deprivation with vasopressin test of the proband*

	Urine volume (L/d)	Basal urinary osmolality (mosmol/L)	Basal Plasma osmolality (mosmol/L)	urinary osmolality after water deprivation (mosmol/L)	plasma osmolality after water deprivation (mosmol/L)	urinary osmolality after injecting vasopressin (mosmol/L)	plasma osmolality after injecting vasopressin (mosmol/L)
Proband	7~10.9	90	299	121	318	154	327

* The dosage of vasopressin injected was 5 u.

The proband had a young cousin (III-2), who was 15 years old and had also presented severe polydipsia and polyuria since the age of 6 months. He was diagnosed NDI by local hospital when he was 15 years old, and the polyuria was reduced significantly by hydrochlorothiazide treatment. All clinical and laboratory investigations and molecular genetics had been conducted in agreement with the family according to Declaration of Helsinki principles. The patients and their family were explained the purpose of the study, and their written informed consents were obtained prior to beginning the study. The research protocol and consent forms were approved by the Reviewer Board and Ethics Committee of Medicine College of Shanghai Jiaotong University.

#### AVPR2 gene analysis

Genomic DNA samples from the two patients and the other eight related normal persons were extracted from the peripheral blood leucocytes using the Flexi Gene DNA Kit (QIAGEN, Hilden, Germany) according to the manufacturer's protocol. Five overlapping primers were designed by software of Primer 3 and were used to amplify the entire coding sequence and their flanking intronic sequences of AVPR2 gene by PCR. The five oligonucleotide primer pairs are as following: 1F: 5' GGA GTT CTG CGT GTC TGT CTG 3', 1R: 5' AGA CAC ACC CAG AGG TGA GG 3', 2F: 5' CTC TGC TAG GAG CCA GGA AGT 3', 2R: 5' GCC AGG ATC ATG TAG GAG GAG 3', 3F: 5' GTG GCT CTG TTC CAA GTG CT 3', 3R: 5' CAC CAG ACT GGC ATG AAT CTC 3', 4F: 5'GAC TGC TGG GCC TGC TTT3', 4R: 5' CCA GCA ACA TGA GTA GCA CAA 3', 5F: 5' ACC TCT GGA AGG TGG GTG TAG 3', 5R: 5' TGT CCA GGG CCA CAC AGT 3'. In addition, the primers used for deletion analysis are shown in Table 2.

**Table 2:** The Primers used for deletion analysis

Primer*	**Sequence (5'→3')**	**Location^#^**	**Amplified regions**	**TM (°C)**	**PCR product size (bp)**
6F	CCTGGCACAGTTCCATGTAGT	3988533-3988513	Intergenic region between L1CAM and AVPR2 genes	61.86	299
6R	TGAGCAGACAACCATCTCCTT	3988811-3988791		61.70	

7F	CCTCGTCCTTGGCTGCCTCCTCTTT	3987895-3987871	Intergenic region between L1CAM and AVPR2 genes	55.00	299
7R	CCGGTTCCCCATGATCAGAACCTT	3988169-3988146		57.14	

8F	CATGTTCTTGGGCTCTCTCCT	3994343-3994363	Intron 21 of the C1 gene	62.55	361
8R	CAGGGCTGAGTTAAGGCTGTT	3994683-3994703		62.49	

9F	GGTGTCTTCTGTCCCTCTCAT	3994750-3994770	Intron 21 of the C1 gene	60.03	273
9R	AGAGCAACACGTGGAGGTGGATAAG	3994998-3995022		61.86	

* F, Forward Primer; R, Reverse Primer

^# ^GeneBank accession number NT_000054 for the AVPR2 gene, as well as upstream and downstream sequences.

The AVPR2 gene was amplified by standard PCR, which consisted of a 25 μl reaction mixture containing 20 ng genomic DNA as template, 8 pmol of each primer, 0.75 mM of each deoxynucleoside triphosphate, 5 mM of MgCl_2_, 1× PCR buffer and 1.0 u of thermal stable Taq Polymerase. The PCR was performed with an initial denaturation at 95°C for 2 minutes, followed by 35 cycles of denaturing at 94°C for 40 seconds, annealing at 58°C for 40 seconds and extending at 72°C for 40 seconds, and then a final extension at 72°C for 10 minutes. PCR products were identified by 1.5% agarose gel electrophoresis and then were purified by shrimp alkaline phosphatase (SAP) and exonuclease I (Exon I)[16]. The purified PCR products were directly sequenced according to standard methodology on an ABI 3700 DNA automated sequencer (Applied Biosystems, CA, USA).

In order to ascertain the breakpoint region, further primers were designed by Primer 3 to amplify flanking sequence of AVPR2. The sequences of primers are listed in Table 2. Genomic DNAs of patient III-1 and III-2 were used as template in PCR with the designed primers and PCR conditions were as above described. To position the deletion breakpoint, a long-PCR was carried out to amplify the region spanning the breakpoint from the genomic DNAs of patient III-1 and III-2. Amplification reactions were performed in a total volume of 25 μl containing 0.3 μM of each deoxynucleoside triphosphate, 40 mM Tricine-KOH (pH 8.7 at 25°C), 15 mM KOAc, 3.5 mM Mg(Oac)_2_, 3.75 μg/ml BSA, 0.005% Tween 20, 0.005% Nonidet-P40, 5 μM of each primer, 80 ng of DNA and 1× advantage™ 2 Polymerase (Clontech, Palo Alto, USA). Cycling conditions were as follows: 94°C for 3 min, followed by 30 cycles of 94°C for 30s, 64°C for 40s and 68°C for 80 seconds, followed by 68°C for 10 min. Long-PCR products were purified by shrimp alkaline phosphatase (SAP) and exonuclease I (Exon I)[16], and then sequenced in both directions on ABI 3700 DNA automated sequencer according to the Big-Dye chemistry reaction protocol (Applied Biosystems, CA, USA).

## Authors' contributions

YD participated in the design of the study and performed PCR, sequencing, data analysis and manuscript preparation. HS participated in the data analysis and manuscript preparation. XC participated in PCR and sequencing. JY participated in the design of the study. QS was responsible for the DNA samples and patients clinical data collection and participated in manuscript preparation. All authors read and approved the final manuscript.
